# Molecular mechanisms of neuroinvasion by monocytes-macrophages in HIV-1 infection

**DOI:** 10.1186/1742-4690-7-30

**Published:** 2010-04-07

**Authors:** Gabriel Gras, Marcus Kaul

**Affiliations:** 1 Institute of Emerging Diseases and Innovative Therapies, Division of Immuno-Virology, CEA, 18 Route du Panorama, F92265 Fontenay-aux Roses, France; 2 Infectious & Inflammatory Disease Center, Burnham Institute for Medical Research, 10901 North Torrey Pines Road, La Jolla, CA 92037, USA

## Abstract

HIV associated neurocognitive disorders and their histopathological correlates largely depend on the continuous seeding of the central nervous system with immune activated leukocytes, mainly monocytes/macrophages from the periphery. The blood-brain-barrier plays a critical role in this never stopping neuroinvasion, although it appears unaltered until the late stage of HIV encephalitis. HIV flux that moves toward the brain thus relies on hijacking and exacerbating the physiological mechanisms that govern blood brain barrier crossing rather than barrier disruption. This review will summarize the recent data describing neuroinvasion by HIV with a focus on the molecular mechanisms involved.

## Introduction

HIV-1 infection is often associated with neurocognitive impairment and the various degrees of severity have recently been categorized under the overarching term HIV associated neurocognitive disorders (HAND) [1]. HAND defines three categories of clinical disorders according to standardized measures of dysfunction: i) asymptomatic neurocognitive impairment (ANI), ii) mild neurocognitive disorder (MND) and iii) HIV-associated dementia (HAD) [2].

HAD constitutes the most severe form of HAND [1] which presented itself prominently at the beginning of the AIDS epidemic but primarily in patients with low CD4+ cell counts and advanced HIV disease [3]. Introduction of combination anti-retroviral therapy (cART)/highly active antiretroviral therapy (HAART) in the mid 1990 improved treatment of HIV infection and often prevented or at least delayed the progression to AIDS and HAD. In recent years, however, and since HIV patients live longer, the incidence of dementia as an AIDS-defining illness has increased, and HAD now defines a significant independent risk factor for death due to AIDS [4,5]. While in the HAART era MND appears to be more prevalent than frank dementia, it appears important to take these long lasting disorders into account in patients' follow up as they may profoundly affect quality of life, complicate autonomy, modify treatment compliance and induce a high level of vulnerability. Moreover, clinical observations over more than 10 years also suggest that HAART cannot completely protect from HAD [1,4-7]. In addition, it is possible that life-long treatment with HAART itself generates a toxicological problem which may affect neurocognitive performance on its own [5,8].

The neuropathological correlates of HIV-1 infection are generally referred to as HIV encephalitis (HIVE) and comprise microglial nodules, activated resident microglia, multinucleated giant cells, infiltration predominantly by monocytoid cells, including blood-derived macrophages, widespread reactive astrocytosis, myelin pallor, and decreased synaptic and dendritic density in combination with distinct neuronal loss [9-11]. HIV-1 associated neuronal damage and loss have been reported for numerous regions of the central nervous system (CNS), including frontal cortex [12,13], substantia nigra [14], cerebellum [15], and putamen [16].

The neuropathology of HIV infection and AIDS has changed under the influence of HAART [6,7,17]. Neuroinflammation was commonly observed in HIV patients at the beginning of the AIDS epidemic and before the introduction of HAART, and usually increased throughout the progression of infected individuals from the latent, asymptomatic stage of the disease to AIDS and HAD [18]. Surprisingly, neuroinflammation seems to persist or even flourish since the advent of HAART [17,19]. Autopsy studies in recent years found microglial activation comparable to that in fully developed AIDS cases from the pre-HAART era, although the primary sites of neuroinflammation are seemingly changed. During pre-HAART times a strong involvement of the basal ganglia was observed whereas post-HAART specimen displayed prominent signs of inflammation in the hippocampus and adjacent parts of the entorhinal and temporal cortex [17]. Interestingly, HAART appeared to limit or even prevent lymphocyte infiltration into the CNS with the exception of the occasionally occurring immune reconstitution inflammatory syndrome (IRIS), that is characterized by massive lymphocytosis, extensive demyelination and white matter damage [6,17].

HIV-1 enters the brain early in the course of infection, presumably via infected macrophages and lymphocytes, and then persists primarily in perivascular macrophages and microglia [11,20,21]. The pathophysiological relevance of CNS invading lymphocytes in HAND remains to be established [22], but CD8+ T cells have been suggested to control intrathecal HIV replication [23]. In contrast to lymphocytes, an increased number of microglia and macrophages correlates well with the severity of pre-mortem HAND [11,18,24].

Infection of the CNS by HIV-1 can be detected and monitored by measurement of viral RNA in cerebrospinal fluid (CSF). Several groups have reported a positive correlation between CSF viral load and the observed degree of cognitive dysfunction in patients with HAND [25-27]. Moreover, CSF viral load appears to correlate with viral load in brain measured by quantitative PCR [27,28], and the highest concentrations of virus are observed in those subcortical structures most frequently affected in patients with severe HAND/HAD [28].

However, in addition to initial neuroinvasion and infection of perivascular macrophages and microglia, factors associated with progressive HIV infection in the periphery, thus outside the brain, may be required to eventually trigger the development of HAND and dementia [29]. One such factor could be an elevated number of circulating monocytes expressing two markers of activated monocytes, CD16 and CD69. Another important player may be the blood brain barrier (BBB) which separates the CNS from the periphery and supposedly controls the traffic of low-molecular-weight nutrients, peptides, proteins and cells in and out of the brain (see for BBB review [30]). Thus the condition of the BBB may potentially determine continuing or repeated neuroinvasion during the course of HIV disease. However, the molecular mechanisms underlying HIV neuroinvasion are only slowly emerging. This review will discuss recent progress in studies of cellular and molecular factors affecting HIV neuroinvasion and consequent neurocognitive sequelae.

## Peripheral Factors Influencing HIV-1 Neuroinvasion

While interferons (IFNs) are important for an anti-viral immune response, the lasting production of IFN-α and -γ in HIV-1 infection has been linked to an erroneous and exhaustive immune activation leading eventually to immune suppression and progression to AIDS [31-33]. In addition, the sustained presence of IFN-α in the HIV-infected CNS correlates with neurocognitive impairment [34,35]. Therefore, IFNs appear to have indeed a major impact on the overall course of HIV disease and consequently also on the development of HAND. However, it is not well understood whether or not IFNs directly influence neuroinvasion of HIV-1. One possible effect may be the IFN-induced expression in the human BBB of APOBEC3G, which has been suggested to account for the limited ability of human brain microvascular endothelial cells (HBMEC) to support HIV-1 replication and thus dissemination into the central nervous system [36].

Peripherally circulating, activated CD16+CD69+ monocytes are prone to adhere to normal endothelium of the brain microvasculature; they transmigrate and might subsequently trigger a number of deleterious processes [29]. Moreover, CD16+ monocytes become an expanding immune cell population during HIV infection [37], particularly with progression to AIDS [38]. These CD16+ monocytes are also more susceptible to HIV infection than the CD16- subset and are the major HIV reservoir among monocytes *in vivo *[39,40]. In fact, CD16+ monocytes likely serve as a vector for HIV trafficking from the periphery into the brain [29,41]. Indeed, although most monocytes do not actively replicate the virus, the macrophages that differentiate from these infected monocytes likely produce large amounts of virus after they quit the circulation, considering that differentiated macrophages are more prone to replicating HIV than monocytes [42-48]. Furthermore, CD16+ monocytes/macrophages can support HIV replication in T-lymphocytes [49] and may be sequestered by tissues expressing the δ-chemokine Fractalkine (Fkn/CX_3_CL1), which include the brain besides lymph nodes and intestine [50-52]. These activated monocytes that represent a latent provirus reservoir in the blood [40] thus may continuously re-seed the brain with infected macrophages and microglia. In addition, macrophages and microglia do replicate HIV in the brain [11,20,21,53] and are not susceptible to the virus' cytopathic effects [54,55] thus permitting them to produce virions throughout their long life span [56-58].

In both HIVE and simian immunodeficiency virus encephalitis (SIVE), CD163^+^/CD16^+ ^macrophages are detected in the parenchyma of the brain and seem to represent the primary productively infected cell population [53]. The elevated number of CD163^+^/CD16^+ ^monocytes/macrophages may reflect an alteration of peripheral mononuclear cell homeostasis and is associated with increased viral burden and reduction of CD4+ T cells. In SIV infection increased viral burden is associated with development of encephalitis, and suggests that the CD163^+^/CD16^+ ^monocyte/macrophage subset may be important in HIV/SIV-associated CNS disease [53]. The critical role of macrophages in the HIV-infected brain is further supported by the viral coreceptor usage. CCR5 is the main coreceptor for HIV infection of macrophages and microglia [59-61], and most virus isolates found in the brain or the CSF use CCR5 [60,62-68]. Of note, the very rare brain-derived R5X4 isolates exhibit tissue specific changes in the V3 region of gp120 that increase the efficiency of CCR5 usage and enhance their tropism for macrophages and microglia [69]. Moreover, macrophage tropism rather than R5 tropism appears to predict neurotropism [67], further emphasizing the role of these cells in NeuroAIDS.

One recent study used fluorescein-positive monocytes in acute simian immunodeficiency virus infection to track neuroinvasion [70]. In this study employing rhesus macaques, fluorescein dye-labeled autologous leukocytes were introduced in the periphery from where the cells subsequently entered into the choroid plexus stromata and perivascular locations in the cerebra during acute SIV infection. The infiltrated cells displayed both CD16 and CD68, both markers for macrophages and microglia. The neuroinvasion of monocytes occurred simultaneously with detectable amounts of virus in CNS tissue and CSF. Furthermore, neuroinvasion was accompanied by the appearance of the proinflammatory chemokines CXCL9/MIG and CCL2/MCP-1 in the brain. Interestingly, before neuroinvasion became obvious, plasma viral load peaked; counts of peripheral blood monocytes rapidly increased; and circulating monocytes displayed an elevated capacity to generate CCL2/MCP-1. Acute infiltration of monocytes into the brain is thus central in early neuroinvasion in the SIV animal model of AIDS. Besides a prominent role of migratory monocytes for SIV/HIV neuroinvasion, this study suggested that a disturbance occurs at the barriers between blood and brain parenchyma as well as blood and CSF [70].

As an alternative to HIV entry via infected macrophages, it has been suggested that the inflammatory cytokine TNF-α promotes a para-cellular route for the virus across the BBB [71]. However, in a study in the feline immunodeficiency virus model, cell-free FIV crossed the BBB only in very low quantities [72]. Moreover, the presence of TNF-α did not change viral transfer or compromise BBB integrity. In contrast, FIV readily crossed the BBB when cell-associated, yet without any significant impairment of the BBB. In response to TNF-α, the migratory activity of uninfected and infected lymphocytes increased in association with an up-regulation of vascular endothelial adhesion molecule (VCAM)-1 and some detectable disturbance of the BBB. Interestingly, once infected cells and TNF-α were introduced on the abluminal side of the BBB in the brain parenchyma, an additional enhanced cell infiltration and more pronounced disruption of the BBB ensued. Moreover, the same study concluded that CNS invasion of lymphocyte-tropic lentiviruses is essentially very similar to that of macrophage-tropic strains [72].

HIV-1 infection compromises the structural integrity of the intestinal tract and can cause leakage of bacteria into the blood stream. Such microbial translocation results in elevated plasma levels of bacterial lipopolysaccharide (LPS), and in HIV-infected/AIDS patients, is associated with increased monocyte activation and dementia [73-75]. Another study suggests that HIV infection increases the vulnerability of the BBB in response to LPS and facilitates the transmigration of peripheral monocytes/macrophages [76]. These findings support an important role for Toll-like receptors (TLRs) besides monocytes and macrophages in HAD [75,76].

On the part of the host, a vicious cycle of immune dysregulation and BBB dysfunction might be required to achieve sufficient entry of infected or activated immune cells into the brain to cause neuronal injury [77,78]. On the side of the virus, variations of the envelope protein gp120 might also influence the timing and extent of events allowing viral entry into the CNS and leading to neuronal injury [79].

## Blood-Brain-Barrier (BBB)

The BBB is widely believed to play an important role in HIV infection of the CNS [29,80]. For example, an acute relapsing brain edema with diffuse BBB alterations and axonal damage was observed early during the AIDS epidemic [81]; and the extravasation of plasma protein through an altered BBB has long been described in AIDS and HIVE cases [82]. *In vivo*, increased permeability of the BBB following HIV/SIV neuroinvasion is associated with the disorganization of tight junctions [83]. In particular zonula occludens (ZO-1) expression is modified in brains of patients with HIV encephalitis [71,84], and loss of occludin and claudin-5 correlates with areas of monocytes infiltration [85]. Such modifications of molecules involved in BBB structure are also found in the brain of SIV-infected macaques with SIVE [86,87]. Nevertheless, these profound modifications of the BBB structure appear to be late events associated with encephalitis whereas neuroinvasion is an early and continuing process.

Regarding the underlying molecular mechanisms involved in BBB crossing by HIV, it appears appropriate to consider in particular the following processes: HIV-dependent cytotoxicity towards cellular BBB components, chemotaxis, regulation of adhesion molecules and tight junction proteins, and last not least the potential influence of drugs of abuse.

### Cytotoxicity Towards Cellular BBB Components

The HIV envelope protein gp120 apparently can trigger cytotoxicity in human brain microvascular endothelial cells (HBMEC) [88]. The process required the presence of IFN-γ and activation of the p38 mitogen-activated protein kinase (MAPK). Interestingly, gp120-induced cytotoxicity occurred only in HBMEC from children but not from adults. The treatment with IFN-γ resulted in an up-regulation of the chemokine receptors CCR3 and CCR5 in HBMECs which in turn may have enhanced the toxic interaction with the viral envelope protein [88].

Interestingly, alterations in the BBB occur even in the absence of intact virus in transgenic mice expressing the HIV envelope protein gp120 in a form that circulates in plasma [89]. This finding suggests that circulating virus or envelope proteins may provoke BBB dysfunction at least during the viremic phase of primary infection.

### Chemotaxis

Neurons, astrocytes and microglia all produce chemokines - cell migration/chemotaxis inducing cytokines - such as monocyte chemoattractant protein CCL2/MCP-1 and CX_3_CL1/Fkn, which appear to attract peripheral blood mononuclear cells (PBMC) across the BBB into the brain parenchyma [22,90].

In fact, an increased risk of HAD has recently been connected to a mutant MCP-1 allele that causes increased infiltration of mononuclear phagocytes into tissues [91]. In HIV/SIV infection, macrophages/microglia and astrocytes express increased quantities of MCP-1/CCL2 [92-94], a chemokine that efficiently attracts monocytes across the BBB. Numerous cell types, including macrophages/microglia, astrocytes and endothelial cells, produce MCP-1 in response to inflammatory stimulation [95]. Of note, HIV infection of macrophages increased their expression of the CCL2 receptor, CCR2, and CCL2 mediated transmigration of HIV-infected PBMC reduced tight junction proteins occludin, claudin-1 and ZO-1 expression in a BBB model *in vitro *[94]. Studies by numerous groups suggested CCL2 in the CNS as a key molecule for HIV encephalitis [96-100] during which it accumulates in the CSF and brain parenchyma [97,101]. Macaques with SIVE behave similarly [100,102,103]. Of importance in HIV infection [96] as well as in the SIV model [100] is that the CCL2 concentration rises in the CSF before neurological signs of the disease occur, conferring to the concentration of CCL2 a potentially prognostic value.

In a mouse model of HIVE based on animals with severe combined immunodeficiency (HIVE-SCID model), HIV-infected microglia and astrocytes seemed to regulate monocyte migration across the BBB via the release of β-chemokines [104]. On the other hand, stromal cell-derived factor (SDF)-1/CXCL12, an α-chemokine, has also been found to influence migration of monocytes by regulating attachment of the cells to HBMEC via the β2 integrin lymphocyte function-associated antigen (LFA)-1 in a Lyn kinase dependent fashion [105]. CXCL12 is up-regulated in neuroinflammatory diseases such as HAND/HAD or multiple sclerosis, and the same study found that the α-chemokine concomitantly reduced monocyte adherence to intercellular adhesion molecule (ICAM)-1, which binds β2 integrins. Interestingly, CXCL12 also counteracted the effect of TNF-α, IL-1β and HIV gp120 regarding an increase of monocyte attachment to HBMEC due to an up-regulation of ICAM-1 [105]. In line with these observations and important for the better understanding of HIV-CNS disease, we found that nerve growth factor (NGF) promotes the attraction of monocytes by CXCL12 with a preferential effect on the CD16+ subset [106], while at the same time decreasing HIV-1 replication in the attracted and infected cells [107], suggesting a specific attraction of uninfected monocytes.

Using an *in vitro *model of the BBB comprised of endothelial cells and astrocytes, another study found that both CXCL12 and CCL2 promoted transmigration of uninfected monocytes and lymphocytes [108]. This investigation also revealed that HIV-1 transactivator of transcription (Tat) induced adhesion molecules and chemokines in astrocytes and microglia which may further increase the trafficking of PBMC into the brain. At the cellular level of monocytes and macrophages, the pro-migratory effect of CCL2 appears to involve K+ channels [109].

A recent microarray study of HBMEC co-cultured with HIV-infected macrophages found the induction of numerous pro-inflammatory and IFN-inducible genes in comparison to endothelial cells exposed to uninfected immune cells [110]. In a separate investigation by the same group, HIVgp120 was observed to trigger in HBMEC the activation of signal transducer and activator of transcription (STAT)-1 and the release of interleukin (IL)-6 and IL-8 [111]. The eukaryotic interleukins and the viral gp120 promoted, in an *in vitro *BBB model, the attachment and transmigration of monocytes; and those processes were prevented by inhibitors of MAPKs, phosphatidyl-inositol 3 kinase (PI3K) or STAT-1 [111]. Furthermore, the pro-inflammatory and IFN-inducible gene products released by HBMEC upon exposure to HIV-1 have been found to down-regulate the expression of tight junction proteins claudin-5, ZO-1, and ZO-2 [112]. Interestingly, an increase of active STAT1 and a reduction of claudin-5 were also found in microvessels of brain specimens from HAD patients [112]. Of note, the HIV-1 envelope protein gp120 seems to be able to trigger many of the effects leading to a compromised BBB and enhanced monocyte transmigration [113].

In line with the altered gene expression of HBMEC exposed to HIV-1 infected macrophages, a proteomic study found that over 200 proteins were up-regulated under the same conditions [114]. The affected cellular components included metabolic pathways, ion channels, cytoskeletal, heat-shock, calcium-binding and transport-related proteins.

Translocation of bacterial LPS from the intestine in HIV-1 infection may not only promote the capability of peripheral monocytes to transmigrate into the brain, but may also encounter a BBB weakened by the effects of a systemic lentiviral infection. In a transgenic mouse model, JR-CSF/EYFP mice, expressing both a long terminal repeat-regulated full-length infectious HIV-1 provirus (JR-CSF) and a ROSA-26-regulated enhanced yellow fluorescent protein (EYFP) as transgenes, peripheral monocytes had an increased capability to enter the brain through an intact or partially compromised BBB [76]. Partial impairment of the BBB was induced by systemic LPS. Importantly, the BBB of JR-CSF/EYFP mice seemed more susceptible to disturbance by LPS than the BBB of HIV-1 free control animals. An earlier *in vitro *study by others found that placing LPS-stimulated macrophages on an artificial BBB led to the occurrence of gaps between endothelial cells and caused a significant increase in monocyte transmigration [115]. The activated monocytes released TNF-α, IL-6 and IL-10, but viral infection itself surprisingly did not increase transmigration under these conditions, suggesting that the LPS exerted a dominant effect. A more recent study found an alternate mechanism where LPS enhanced the trans-cellular transport of HIV-1 across the BBB via a p38 MAPK-dependent pathway [116].

Tryptophan metabolism via the kynurenine pathway occurs in the human BBB during HIV-1 infection and has been linked to immune tolerance and neurotoxicity [117]. Endothelial cells and pericytes of the BBB, as well as astrocytes [118], acquire upon immune stimulation the capability to produce kynurenine, which when released into the vicinity of macrophages and microglia could be further metabolized to the neurotoxin quinolinic acid [119]. Of note, IFNs and LPS are both able to activate tryptophan catabolism in macrophages [120], a process that may add to the effects of BBB activation during HIV infection. Thus, peripheral HIV-1 infection and associated immune stimulation side by side with LPS translocation could potentially exert neurotoxicity across the BBB even without the virus entering the brain.

### Adhesion Molecules

Cell migration also engages adhesion molecules, and increased expression of various adhesion molecules, such as VCAM-1, has been implicated in mononuclear cell migration into the brain during HIV and SIV infection [80,115,121,122]. Astrocytes apparently control expression of ICAM-1 in endothelial cells of the BBB, and upon exposure to TNF-α, produce themselves ICAM-1, VCAM-1, IG9 and E-selectin, all of which may promote monocyte attachment and transmigration [121].

HIV-infected macrophages, in particular when additionally stimulated with LPS, induce expression of E-selectin and VCAM-1 in brain microvascular endothelial cells (BMEC) [80]. In brain specimens from AIDS patients with HIVE, detection of E-selectin and VCAM-1 correlated with HIV-1 and pro-inflammatory cytokines; and an association of invading macrophages and increased signal for endothelial adhesion molecules were observed in HIVE samples.

Possibly counteracting the effects of pro-inflammatory cytokines, the activation of peroxisome proliferator-activated receptor γ (PPARγ) in HBMECs can suppress the activity of Rho GTPases (Rac1 and RhoA) and inhibit adhesion and transendothelial migration of HIV-1 infected monocytes [123].

### Tight Junction Proteins

Concomitant with the development of HIVE, the expression of tight junction proteins between BMECs of the BBB decreases. The disruption of tight junctions between BMECs is apparently mediated through the activation of focal adhesion kinase (FAK) by phosphorylation at TYR-397 [124]. Furthermore, HIV-1 gp120 seems capable of inducing the disruption of tight junctions by triggering proteasomal degradation of ZO-1 and ZO-2 in HBMEC [125]. Interestingly, the scaffolding protein 14-3-3tau appears to counteract the down-regulation by HIV gp120 of ZO-1 and ZO-2; and even more surprisingly, the viral envelope protein specifically increases expression of 14-3-3tau [125].

In addition to HIV gp120, Tat also affects tight junction proteins [126]. As such Tat reduces the expression of occludin, ZO-1, and ZO-2 in the caveolar compartment of HBMECs. The effect of Tat is dependent on caveolin-1 and its modulation of Ras signaling.

### Drugs of Abuse and Alcohol

Abuse of psycho-stimulatory and addictive drugs seems to increase the risk of HIV-1 infection and of the development of HAND [127-130].

HIV Tat and morphine apparently cooperate in diminishing the electrical resistance and increasing the transmigration across the BBB via the activation of pro-inflammatory cytokines, the stimulation of intracellular Ca^2+ ^release, and the activation of myosin light chain kinase [131]. A similar effect is caused by both methamphetamine and HIV gp120 either alone or in combination [132].

Cocaine also alters the expression of tight junction proteins and induces stress fibers in BMECs, and it in addition up-regulates the pro-migratory CCL2/CCR2 ligand-receptor system thus facilitating the passage of HIV-infected monocytes through the BBB [133]. In an *in vitro *BBB model comprising endothelial cells and astrocytes, cocaine was also found to decrease barrier function, increase expression of ICAM-1, VCAM-1 and platelet-endothelial cell adhesion molecule (PECAM)-1, and to enhance monocyte migration across the BBB [134].

In contrast to the before-mentioned drugs, cannabinoids have been reported to preserve in HBMECs, in the presence of HIV gp120, the expression of tight junction proteins. Cannabinoids decrease the permeability of the BBB and inhibit the transmigration of HIV-infected monocytes through the barrier [135].

Alcohol and HIV-1 gp120 both affect BBB permeability and stress fiber formation in BMECs [136]. Interestingly, all these effects can apparently be ameliorated by the inhibition of reactive oxygen species [136].

## General considerations and conclusion

**Figure 1:**
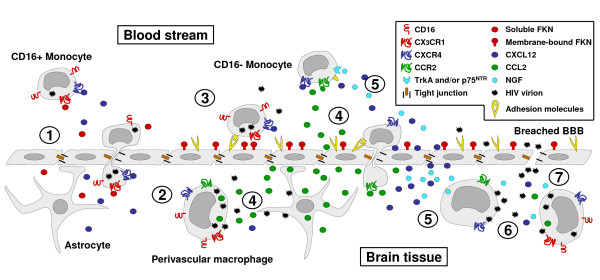
**Mechanistic model of HIV-1 neuroinvasion**. **(1) **The physiological expression of chemokines by brain cells, among which are soluble fractalkine (Fkn) and CXCL12, supports a slow but continuous entry of monocytes and macrophages into the central nervous system. Due to their expression of CX_3_CR1, CD16 positive, activated monocytes are the preferential targets for such attraction. These CD16 positive monocytes are the main reservoir of monocyte/macrophage-harbored virus and are thus likely to be the predominant cell type carrying HIV into the brain. **(2) **Infiltrated HIV-infected monocytes locally produce HIV and inflammatory mediators in perivascular areas. This activates neighbouring astrocytes as well as the blood brain barrier (BBB) endothelium. **(3) **In response, endothelial cells up-regulate adhesion molecules, enhancing monocyte recruitment. However, membrane-bound Fkn is also induced on endothelial cells and can arrest CD16 positive monocytes at the endothelium thus inhibiting their further infiltration. **(4) **CCL2 is overexpressed by infected, HIV-stimulated macrophages and activated astrocytes, attracting CD16 negative, CCR2 positive monocytes toward the perivascular area. **(5) **Both CXCL12 and nerve growth factor (NGF) are overexpressed in the inflamed brain. NGF increases CXCR4 expression and promotes uninfected monocyte attraction by CXCL12. At the same time it limits entry of infected monocytes into the brain. **(6) **Activated uninfected perivascular macrophages may be targets for *de novo *infection by locally produced HIV, amplifying the activation - attraction - infection cycle. **(7) **Local inflammation as well as HIV products induce tight junction disorganization and lead to breaches in the BBB. Toxic serum proteins and free virions may enter the brain, favouring more infection and further amplifying inflammation.

HIV enters the CNS very early after infection, and then maintains its presence in the brain throughout the individual's life. Interestingly, major alterations of the BBB occur only late in HIV-CNS disease and thus initial seeding likely reflects the hijacking of physiological mechanisms of BBB crossing, such as the Trojan horse strategy initially proposed by Narayan and colleagues [137,138]. A model of the multistep, multifactorial process of CNS invasion by HIV-1, is illustrated in figure 1. It has for years remained unclear whether the infected CNS constituted, after its initial seeding, a viral sanctuary independent of the periphery or just reflected infection features outside the brain. The introduction of HAART challenged our vision of the brain as an independent sanctuary of HIV infection because the lower incidence of HAD in treated patients, despite low brain penetration of the molecules, strongly suggested that HIV induced CNS disorders do require continuous immune activation in the brain and neuroinvasion of activated and/or infected leukocytes.

This interdependence is exemplified by the fact that, in humans and in animal models, neurological complications of HIV infection correlate not only with innate immunity [35] and macrophage/microglia activation [11,18,24] within the brain tissue, but also with proviral load in activated peripheral CD16+ monocytes/macrophages [29,40,41]. In this context, BBB crossing by HIV infected and immune-activated macrophages appears to be a critical target for future therapeutic developments. The very complex and intricate mechanisms that govern this crossing should thus be studied with particular attention.

HAND correlate with CSF viral load [25], which is closely related to CSF pleocytosis [139]. In a recent study, Sinclair et al. showed that HAART despite treatment failures with no effect on peripheral viral load, had nevertheless a significant beneficial impact on CSF viral load, CSF pleocytosis, and immune activation [140]. This striking and encouraging result further illustrates the critical importance of an improved understanding of BBB function and neuroinvasion mechanisms. Furthermore, HIV neuroinvasion and BBB likely will provide future therapeutic targets for coping with the anticipated increase in HAND prevalence as more and more HIV patients come of age.

## Competing interests

The authors declare that they have no competing interests.

## Authors' contributions

GG and MK wrote the article jointly. All authors read and approved the final manuscript.
